# *N*-(2,3-Dimethyl­phen­yl)-4-hydr­oxy-2-methyl-2*H*-1,2-benzothia­zine-3-carboxamide 1,1-dioxide

**DOI:** 10.1107/S1600536809006837

**Published:** 2009-02-28

**Authors:** Waseeq Ahmad Siddiqui, Iftikhar Hussain Bukahari, Muhammad Zia-ur-Rehman, Islam Ullah Khan, Graham John Tizzard

**Affiliations:** a Department of Chemistry, University of Sargodha, Sargodha, Pakistan; b Applied Chemistry Research Centre, PCSIR Laboratories Complex, Ferozpure Road, Lahore 54600, Pakistan; c Department of Chemistry, Government College University, Lahore 54000, Pakistan; d School of Chemistry, University of Southampton, England

## Abstract

In the crystal structure of the title compound, C_18_H_18_N_2_O_4_S, the thia­zine ring adopts a distorted half-chair conformation. 1,2-Benzothia­zines of this kind have a wide range of biological activities and are mainly used as medicines in the treatment of inflammation and rheumatoid arthritis. The enolic H atom is involved in an intra­molecular O—H⋯O hydrogen bond, forming a six-membered ring. The mol­ecules arrange themselves into centrosymmetric dimers by means of inter­molecular N—H⋯O hydrogen bonds. A weak inter­molcular C—H⋯O inter­action is also present.

## Related literature

For the synthesis of related mol­ecules, see: Siddiqui, Ahmad, Khan *et al.* (2007 ▶); Zia-ur-Rehman *et al.* (2006 ▶); For the biological activity of 1,2-benzothia­zine-1,1-dioxides, see: Zia-ur-Rehman *et al.* (2009 ▶). For related structures, see: Siddiqui *et al.* (2008 ▶); Siddiqui, Ahmad, Siddiqui *et al.* (2007 ▶). For the pharmacological background to 1,2-benzothia­zine-3-carboxamide 1,1-dioxide derivatives, see Gennari *et al.* (1994 ▶); Lombardino & Wiseman (1972 ▶).
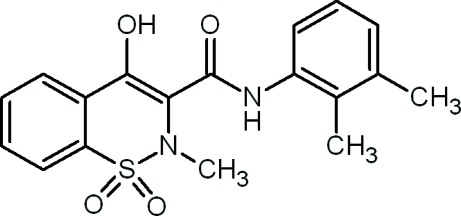


## Experimental

### 

#### Crystal data

C_18_H_18_N_2_O_4_S
*M* *_r_* = 358.40
Monoclinic, 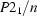

*a* = 10.2461 (3) Å
*b* = 8.5421 (2) Å
*c* = 19.8944 (5) Å
β = 104.832 (1)°
*V* = 1683.20 (8) Å^3^
*Z* = 4
Mo *K*α radiation
μ = 0.22 mm^−1^
*T* = 120 K
0.30 × 0.10 × 0.10 mm

#### Data collection

Bruker–Nonius CCD camera on κ-goniostat diffractometer
Absorption correction: multi-scan (*SADABS*; Sheldrick, 2007 ▶) *T*_min_ = 0.855, *T*_max_ = 0.978
18371 measured reflections
3828 independent reflections
3000 reflections with *I* > 2σ(*I*)
*R*_int_ = 0.055

#### Refinement

*R*[*F* ^2^ > 2σ(*F* ^2^)] = 0.045
*wR*(*F* ^2^) = 0.114
*S* = 1.06
3828 reflections
234 parameters
H atoms treated by a mixture of independent and constrained refinement
Δρ_max_ = 0.27 e Å^−3^
Δρ_min_ = −0.57 e Å^−3^

### 

Data collection: *COLLECT* (Hooft, 1998 ▶); cell refinement: *DENZO* (Otwinowski & Minor, 1997 ▶) and *COLLECT*; data reduction: *DENZO* and *COLLECT*; program(s) used to solve structure: *SHELXS97* (Sheldrick, 2008 ▶); program(s) used to refine structure: *SHELXL97* (Sheldrick, 2008 ▶); molecular graphics: *SHELXTL* (Sheldrick, 2008 ▶); software used to prepare material for publication: *SHELXTL* and local programs.

## Supplementary Material

Crystal structure: contains datablocks I, global. DOI: 10.1107/S1600536809006837/bt2887sup1.cif

Structure factors: contains datablocks I. DOI: 10.1107/S1600536809006837/bt2887Isup2.hkl

Additional supplementary materials:  crystallographic information; 3D view; checkCIF report

## Figures and Tables

**Table 1:** Hydrogen-bond geometry (Å, °)

*D*—H⋯*A*	*D*—H	H⋯*A*	*D*⋯*A*	*D*—H⋯*A*
O1—H1*O*⋯O2	0.84	1.79	2.5320 (18)	147
N2—H1*N*⋯O3^i^	0.85 (2)	2.26 (2)	2.972 (2)	141 (2)
C3—H3⋯O4^ii^	0.95	2.49	3.352 (3)	150

Symmetry codes: (i) 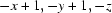; (ii) 
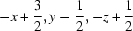
.

**Fig. 1.**
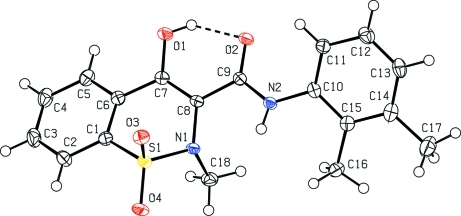
The molecular structure of the title compound with displacement ellipsoids at the 50% probability level.

**Fig. 2.**
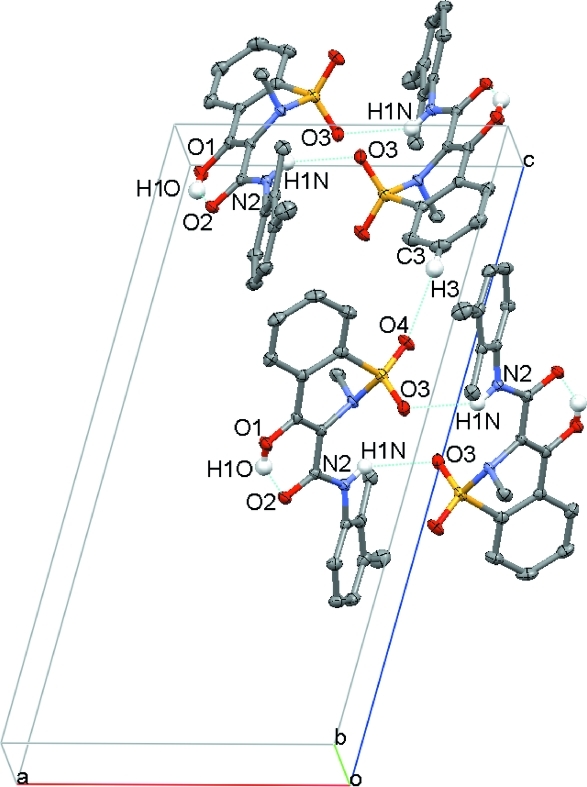
Perspective view of the three-dimensional crystal packing showing hydrogen-bonded interactions (dashed lines). H atoms not involved in hydrogen bonding have been omitted for clarity.

## supplementary crystallographic information

### Comment

1,2-Benzothiazine-3-carboxamide 1,1-dioxide derivatives belonging to oxicams, a class of non-steroidal anti-inflammatory drugs (NSAIDs), are well known as analgesic and anti-inflammatory agents since the introduction of Piroxicam (Lombardino & Wiseman, 1972) in the United States in 1982 where it gained immediate acceptance and remained among the top fifty prescription drugs for several years. Besides having anti-inflammatory activity, these have also been found to be used for the treatment of rheumatoid arthritis, ankylosing spondylitis, osteoarthrosis and other inflammatory rheumatic and non- rheumatic processes, including onsets and traumatologic lesions (Gennari *et al.,* 1994).

In continuation of our work on the synthesis (Siddiqui, Ahmad, Khan  *et al.*, 2007, Zia-ur-Rehman *et al.*, 2006, biological activity (Zia-ur-Rehman *et al*, 2009) and crystal structures (Siddiqui, Ahmad, Siddiqui *et al.*, 2007, Siddiqui *et al.*, 2008) of various 1,2-benzothiazine-1,1-dioxides, we herein report the synthesis and crystal structure of the title compound (I) (Scheme and figure 1). The thiazine ring, involving two double bonds, exhibits a distorted half-chair conformation. The enolic hydrogen on O1 is involved in intramolecular hydrogen bonding giving rise to a six-membered hydrogen bond ring (Table 1). The molecules form centrosymmetric dimers   through intermolecular N—H···O hydrogen bonds. In addition, the crystal packing is stabilized by weak C—H···O contacts.

### Experimental

A mixture of methyl 4-hydroxy-2-methyl-2*H*-1,2-benzothiazine-3-carboxylate-1,1-dioxide (2.693 g; 10.0 mmoles), 2,3-dimethyl aniline (1.818 g; 15.0 mmoles) and xylene (25.0 ml) was refluxed under nitrogen atmosphere in a Soxhlet apparatus having Linde type 4Å molecular sieves. Three fourth of the xylene was then distilled off and the remaining contents were allowed to stand overnight at room temperature. Settled solids were filtered off, washed with diethyl ether and crystallized from ethanol. Yield: 78%.

### Refinement

All hydrogen atoms were identified in the difference map. Those bonded to O and C were  fixed in ideal positions and treated as riding on their parent atoms. In the case of the methyl and hydroxyl H atoms the torsion angles were refined. The following distances were used: Methyl C—H 0.98 Å. ° Aromatic C—H 0.95 Å. ° Hydroxyl O—H 0.84 Å. U(H) was set to 1.2Ueq of the parent atoms or 1.5Ueq for methyl groups. The H atom bonded to N was freely refined.

### Crystal data

**Table:**

C_18_H_18_N_2_O_4_S	*F*(000) = 752
*M**_r_* = 358.40	*D*_x_ = 1.414 Mg m^−^^3^
Monoclinic, *P*2_1_/*n*	Mo *K*α radiation, λ = 0.71073 Å
Hall symbol: -P 2yn	Cell parameters from 10340 reflections
*a* = 10.2461 (3) Å	θ = 2.9–27.5°
*b* = 8.5421 (2) Å	µ = 0.22 mm^−^^1^
*c* = 19.8944 (5) Å	*T* = 120 K
β = 104.832 (1)°	Block, colourless
*V* = 1683.20 (8) Å^3^	0.30 × 0.10 × 0.10 mm
*Z* = 4	

### Data collection

**Table:**

Bruker–Nonius CCD camera on κ-goniostat diffractometer	3828 independent reflections
Radiation source: Bruker Nonius FR591 Rotating Anode	3000 reflections with *I* > 2σ(*I*)
graphite	*R*_int_ = 0.055
Detector resolution: 9.091 pixels mm^-1^	θ_max_ = 27.5°, θ_min_ = 3.2°
φ and ω scans to fill the asymmetric unit	*h* = −13→11
Absorption correction: multi-scan (*SADABS*; Sheldrick, 2007)	*k* = −11→9
*T*_min_ = 0.855, *T*_max_ = 0.978	*l* = −25→24
18371 measured reflections	

### Refinement

**Table:**

Refinement on *F*^2^	Primary atom site location: structure-invariant direct methods
Least-squares matrix: full	Secondary atom site location: difference Fourier map
*R*[*F*^2^ > 2σ(*F*^2^)] = 0.045	Hydrogen site location: inferred from neighbouring sites
*wR*(*F*^2^) = 0.114	H atoms treated by a mixture of independent and constrained refinement
*S* = 1.06	*w* = 1/[σ^2^(*F*_o_^2^) + (0.0495*P*)^2^ + 0.8901*P*] where *P* = (*F*_o_^2^ + 2*F*_c_^2^)/3
3828 reflections	(Δ/σ)_max_ = 0.001
234 parameters	Δρ_max_ = 0.27 e Å^−^^3^
0 restraints	Δρ_min_ = −0.57 e Å^−^^3^

### Special details

**Table:**

Experimental. *SADABS* was used to perform the Absorption correction Parameter refinement on 11612 reflections reduced *R*(int) from 0.0681 to 0.0328 Ratio of minimum to maximum apparent transmission: 0.867291 The given Tmin and Tmax were generated using the *SHELX* SIZE command
Geometry. All e.s.d.'s (except the e.s.d. in the dihedral angle between two l.s. planes) are estimated using the full covariance matrix. The cell e.s.d.'s are taken into account individually in the estimation of e.s.d.'s in distances, angles and torsion angles; correlations between e.s.d.'s in cell parameters are only used when they are defined by crystal symmetry. An approximate (isotropic) treatment of cell e.s.d.'s is used for estimating e.s.d.'s involving l.s. planes.
Refinement. Refinement of *F*^2^ against ALL reflections. The weighted *R*-factor *wR* and goodness of fit *S* are based on *F*^2^, conventional *R*-factors *R* are based on *F*, with *F* set to zero for negative *F*^2^. The threshold expression of *F*^2^ > σ(*F*^2^) is used only for calculating *R*-factors(gt) *etc*. and is not relevant to the choice of reflections for refinement. *R*-factors based on *F*^2^ are statistically about twice as large as those based on *F*, and *R*- factors based on ALL data will be even larger.

### Fractional atomic coordinates and isotropic or equivalent isotropic displacement parameters (Å^2^)

**Table:**

	*x*	*y*	*z*	*U*_iso_*/*U*_eq_	
S1	0.65769 (5)	0.38059 (5)	0.08727 (2)	0.01787 (14)	
O2	0.85778 (13)	0.51574 (15)	−0.10128 (6)	0.0190 (3)	
O1	0.93907 (13)	0.27205 (16)	−0.03046 (7)	0.0208 (3)	
H1O	0.9310	0.3360	−0.0633	0.031*	
N2	0.70234 (16)	0.67387 (18)	−0.06965 (8)	0.0162 (3)	
N1	0.73387 (15)	0.51959 (17)	0.05471 (8)	0.0155 (3)	
O3	0.55860 (13)	0.31248 (15)	0.03050 (7)	0.0207 (3)	
O4	0.61656 (14)	0.44317 (17)	0.14534 (7)	0.0255 (3)	
C6	0.88260 (18)	0.2273 (2)	0.07631 (9)	0.0173 (4)	
C9	0.78894 (18)	0.5530 (2)	−0.05975 (9)	0.0154 (4)	
C10	0.67298 (17)	0.7818 (2)	−0.12596 (9)	0.0166 (4)	
C7	0.87323 (18)	0.3265 (2)	0.01508 (9)	0.0162 (4)	
C1	0.78842 (19)	0.2430 (2)	0.11619 (9)	0.0187 (4)	
C18	0.8113 (2)	0.6356 (2)	0.10495 (10)	0.0226 (4)	
H18A	0.8911	0.5851	0.1348	0.034*	
H18B	0.7542	0.6764	0.1336	0.034*	
H18C	0.8397	0.7220	0.0795	0.034*	
C5	0.98373 (19)	0.1138 (2)	0.09600 (10)	0.0216 (4)	
H5	1.0480	0.1000	0.0695	0.026*	
C8	0.80079 (18)	0.4615 (2)	0.00417 (9)	0.0151 (4)	
C14	0.61045 (19)	1.0447 (2)	−0.16562 (11)	0.0226 (4)	
C16	0.6568 (2)	0.9831 (2)	−0.03662 (10)	0.0233 (4)	
H16A	0.5896	0.9260	−0.0190	0.035*	
H16B	0.6410	1.0959	−0.0344	0.035*	
H16C	0.7475	0.9578	−0.0082	0.035*	
C4	0.9901 (2)	0.0215 (2)	0.15404 (11)	0.0266 (5)	
H4	1.0594	−0.0549	0.1672	0.032*	
C15	0.64525 (18)	0.9363 (2)	−0.11082 (10)	0.0181 (4)	
C12	0.6398 (2)	0.8437 (2)	−0.24637 (10)	0.0246 (4)	
H12	0.6402	0.8140	−0.2923	0.030*	
C11	0.67017 (19)	0.7340 (2)	−0.19320 (9)	0.0203 (4)	
H11	0.6887	0.6283	−0.2025	0.024*	
C3	0.8966 (2)	0.0393 (2)	0.19319 (11)	0.0294 (5)	
H3	0.9024	−0.0246	0.2329	0.035*	
C13	0.6091 (2)	0.9960 (2)	−0.23262 (10)	0.0248 (4)	
H13	0.5865	1.0692	−0.2697	0.030*	
C2	0.7944 (2)	0.1507 (2)	0.17439 (10)	0.0258 (4)	
H2	0.7299	0.1633	0.2009	0.031*	
C17	0.5769 (2)	1.2125 (3)	−0.15299 (12)	0.0350 (5)	
H17A	0.5529	1.2691	−0.1973	0.052*	
H17B	0.6555	1.2624	−0.1217	0.052*	
H17C	0.5006	1.2151	−0.1317	0.052*	
H1N	0.657 (2)	0.683 (3)	−0.0397 (12)	0.026 (6)*	

### Atomic displacement parameters (Å^2^)

**Table:**

	*U*^11^	*U*^22^	*U*^33^	*U*^12^	*U*^13^	*U*^23^
S1	0.0187 (2)	0.0193 (3)	0.0181 (2)	−0.00080 (18)	0.00918 (18)	−0.00048 (18)
O2	0.0210 (7)	0.0221 (7)	0.0160 (6)	0.0015 (5)	0.0085 (5)	0.0000 (5)
O1	0.0218 (7)	0.0229 (7)	0.0208 (7)	0.0045 (6)	0.0112 (6)	0.0014 (5)
N2	0.0159 (8)	0.0182 (8)	0.0155 (8)	0.0018 (6)	0.0062 (6)	0.0012 (6)
N1	0.0162 (8)	0.0170 (8)	0.0151 (7)	−0.0005 (6)	0.0073 (6)	−0.0023 (6)
O3	0.0156 (7)	0.0230 (7)	0.0245 (7)	−0.0022 (5)	0.0072 (5)	−0.0032 (6)
O4	0.0325 (8)	0.0269 (8)	0.0232 (7)	0.0000 (6)	0.0183 (6)	−0.0027 (6)
C6	0.0168 (9)	0.0173 (9)	0.0163 (9)	−0.0034 (7)	0.0018 (7)	−0.0016 (7)
C9	0.0157 (9)	0.0163 (9)	0.0136 (8)	−0.0038 (7)	0.0028 (7)	−0.0017 (7)
C10	0.0121 (8)	0.0192 (9)	0.0170 (9)	−0.0021 (7)	0.0013 (7)	0.0026 (7)
C7	0.0130 (8)	0.0187 (9)	0.0171 (9)	−0.0023 (7)	0.0041 (7)	−0.0018 (7)
C1	0.0223 (9)	0.0172 (9)	0.0161 (9)	−0.0019 (7)	0.0037 (7)	−0.0020 (7)
C18	0.0263 (10)	0.0227 (10)	0.0197 (10)	−0.0050 (8)	0.0075 (8)	−0.0057 (8)
C5	0.0192 (10)	0.0194 (10)	0.0243 (10)	0.0010 (7)	0.0020 (8)	−0.0008 (8)
C8	0.0147 (8)	0.0179 (9)	0.0134 (9)	−0.0015 (7)	0.0049 (7)	−0.0016 (7)
C14	0.0180 (9)	0.0190 (10)	0.0273 (10)	−0.0014 (8)	−0.0003 (8)	0.0024 (8)
C16	0.0267 (11)	0.0192 (10)	0.0250 (10)	0.0004 (8)	0.0083 (9)	−0.0035 (8)
C4	0.0292 (11)	0.0210 (10)	0.0246 (10)	0.0028 (8)	−0.0023 (9)	0.0033 (8)
C15	0.0140 (9)	0.0193 (9)	0.0199 (9)	−0.0028 (7)	0.0025 (7)	−0.0007 (7)
C12	0.0275 (11)	0.0277 (11)	0.0163 (9)	−0.0036 (8)	0.0015 (8)	0.0010 (8)
C11	0.0235 (10)	0.0196 (10)	0.0168 (9)	−0.0011 (8)	0.0034 (8)	−0.0005 (7)
C3	0.0429 (13)	0.0229 (11)	0.0187 (10)	−0.0008 (9)	0.0013 (9)	0.0054 (8)
C13	0.0250 (11)	0.0249 (11)	0.0211 (10)	−0.0024 (8)	−0.0005 (8)	0.0073 (8)
C2	0.0353 (12)	0.0248 (10)	0.0190 (10)	−0.0030 (9)	0.0101 (9)	0.0017 (8)
C17	0.0428 (14)	0.0220 (11)	0.0361 (13)	0.0039 (9)	0.0029 (11)	0.0019 (9)

### Geometric parameters (Å, °)

**Table:**

S1—O4	1.4308 (13)	C5—C4	1.386 (3)
S1—O3	1.4345 (14)	C5—H5	0.9500
S1—N1	1.6424 (15)	C14—C13	1.393 (3)
S1—C1	1.7639 (19)	C14—C15	1.405 (3)
O2—C9	1.257 (2)	C14—C17	1.510 (3)
O1—C7	1.344 (2)	C16—C15	1.504 (3)
O1—H1O	0.8400	C16—H16A	0.9800
N2—C9	1.343 (2)	C16—H16B	0.9800
N2—C10	1.422 (2)	C16—H16C	0.9800
N2—H1N	0.85 (2)	C4—C3	1.389 (3)
N1—C8	1.442 (2)	C4—H4	0.9500
N1—C18	1.485 (2)	C12—C13	1.383 (3)
C6—C5	1.400 (3)	C12—C11	1.388 (3)
C6—C1	1.404 (3)	C12—H12	0.9500
C6—C7	1.467 (3)	C11—H11	0.9500
C9—C8	1.471 (2)	C3—C2	1.394 (3)
C10—C11	1.392 (3)	C3—H3	0.9500
C10—C15	1.399 (3)	C13—H13	0.9500
C7—C8	1.359 (3)	C2—H2	0.9500
C1—C2	1.389 (3)	C17—H17A	0.9800
C18—H18A	0.9800	C17—H17B	0.9800
C18—H18B	0.9800	C17—H17C	0.9800
C18—H18C	0.9800		
			
O4—S1—O3	119.46 (8)	C7—C8—C9	120.66 (16)
O4—S1—N1	108.44 (8)	N1—C8—C9	118.12 (15)
O3—S1—N1	107.14 (8)	C13—C14—C15	118.96 (18)
O4—S1—C1	109.98 (9)	C13—C14—C17	119.71 (18)
O3—S1—C1	108.17 (8)	C15—C14—C17	121.33 (18)
N1—S1—C1	102.26 (8)	C15—C16—H16A	109.5
C7—O1—H1O	109.5	C15—C16—H16B	109.5
C9—N2—C10	127.78 (16)	H16A—C16—H16B	109.5
C9—N2—H1N	115.6 (16)	C15—C16—H16C	109.5
C10—N2—H1N	116.6 (16)	H16A—C16—H16C	109.5
C8—N1—C18	115.56 (14)	H16B—C16—H16C	109.5
C8—N1—S1	112.65 (12)	C5—C4—C3	120.83 (19)
C18—N1—S1	116.30 (12)	C5—C4—H4	119.6
C5—C6—C1	118.36 (17)	C3—C4—H4	119.6
C5—C6—C7	121.28 (17)	C10—C15—C14	118.67 (17)
C1—C6—C7	120.36 (17)	C10—C15—C16	119.51 (17)
O2—C9—N2	123.99 (16)	C14—C15—C16	121.80 (17)
O2—C9—C8	119.87 (16)	C13—C12—C11	120.17 (18)
N2—C9—C8	116.14 (15)	C13—C12—H12	119.9
C11—C10—C15	121.91 (17)	C11—C12—H12	119.9
C11—C10—N2	120.98 (17)	C12—C11—C10	118.68 (18)
C15—C10—N2	117.10 (16)	C12—C11—H11	120.7
O1—C7—C8	122.29 (16)	C10—C11—H11	120.7
O1—C7—C6	114.99 (16)	C4—C3—C2	120.22 (19)
C8—C7—C6	122.71 (16)	C4—C3—H3	119.9
C2—C1—C6	121.79 (18)	C2—C3—H3	119.9
C2—C1—S1	121.16 (15)	C12—C13—C14	121.55 (18)
C6—C1—S1	117.01 (14)	C12—C13—H13	119.2
N1—C18—H18A	109.5	C14—C13—H13	119.2
N1—C18—H18B	109.5	C1—C2—C3	118.73 (19)
H18A—C18—H18B	109.5	C1—C2—H2	120.6
N1—C18—H18C	109.5	C3—C2—H2	120.6
H18A—C18—H18C	109.5	C14—C17—H17A	109.5
H18B—C18—H18C	109.5	C14—C17—H17B	109.5
C4—C5—C6	120.06 (18)	H17A—C17—H17B	109.5
C4—C5—H5	120.0	C14—C17—H17C	109.5
C6—C5—H5	120.0	H17A—C17—H17C	109.5
C7—C8—N1	121.22 (16)	H17B—C17—H17C	109.5
			
O4—S1—N1—C8	169.35 (12)	C6—C7—C8—C9	−176.92 (16)
O3—S1—N1—C8	−60.45 (14)	C18—N1—C8—C7	94.4 (2)
C1—S1—N1—C8	53.19 (14)	S1—N1—C8—C7	−42.6 (2)
O4—S1—N1—C18	32.66 (15)	C18—N1—C8—C9	−85.4 (2)
O3—S1—N1—C18	162.85 (13)	S1—N1—C8—C9	137.60 (14)
C1—S1—N1—C18	−83.51 (14)	O2—C9—C8—C7	−7.7 (3)
C10—N2—C9—O2	−1.0 (3)	N2—C9—C8—C7	172.23 (16)
C10—N2—C9—C8	179.07 (16)	O2—C9—C8—N1	172.09 (16)
C9—N2—C10—C11	35.7 (3)	N2—C9—C8—N1	−8.0 (2)
C9—N2—C10—C15	−145.22 (18)	C6—C5—C4—C3	0.3 (3)
C5—C6—C7—O1	18.7 (2)	C11—C10—C15—C14	1.6 (3)
C1—C6—C7—O1	−160.50 (16)	N2—C10—C15—C14	−177.46 (16)
C5—C6—C7—C8	−162.85 (18)	C11—C10—C15—C16	−176.85 (17)
C1—C6—C7—C8	18.0 (3)	N2—C10—C15—C16	4.1 (2)
C5—C6—C1—C2	0.7 (3)	C13—C14—C15—C10	−2.1 (3)
C7—C6—C1—C2	179.90 (17)	C17—C14—C15—C10	178.91 (18)
C5—C6—C1—S1	−176.97 (14)	C13—C14—C15—C16	176.29 (18)
C7—C6—C1—S1	2.2 (2)	C17—C14—C15—C16	−2.7 (3)
O4—S1—C1—C2	32.47 (18)	C13—C12—C11—C10	−2.0 (3)
O3—S1—C1—C2	−99.61 (17)	C15—C10—C11—C12	0.5 (3)
N1—S1—C1—C2	147.51 (16)	N2—C10—C11—C12	179.45 (17)
O4—S1—C1—C6	−149.86 (14)	C5—C4—C3—C2	0.1 (3)
O3—S1—C1—C6	78.06 (16)	C11—C12—C13—C14	1.4 (3)
N1—S1—C1—C6	−34.82 (16)	C15—C14—C13—C12	0.6 (3)
C1—C6—C5—C4	−0.7 (3)	C17—C14—C13—C12	179.7 (2)
C7—C6—C5—C4	−179.94 (17)	C6—C1—C2—C3	−0.2 (3)
O1—C7—C8—N1	−178.38 (15)	S1—C1—C2—C3	177.33 (16)
C6—C7—C8—N1	3.3 (3)	C4—C3—C2—C1	−0.2 (3)
O1—C7—C8—C9	1.4 (3)		

### Hydrogen-bond geometry (Å, °)

**Table:**

*D*—H···*A*	*D*—H	H···*A*	*D*···*A*	*D*—H···*A*
O1—H1O···O2	0.84	1.79	2.5320 (18)	147
N2—H1N···O3^i^	0.85 (2)	2.26 (2)	2.972 (2)	141 (2)
C3—H3···O4^ii^	0.95	2.49	3.352 (3)	150

Symmetry codes: (i) −*x*+1, −*y*+1, −*z*; (ii) −*x*+3/2, *y*−1/2, −*z*+1/2.
